# Intra-familial and ethnic effects on attitudinal and perceptual body image: a cohort of South African mother-daughter dyads

**DOI:** 10.1186/1471-2458-11-433

**Published:** 2011-06-06

**Authors:** Zandile J Mchiza, Julia H Goedecke, Estelle V Lambert

**Affiliations:** 1 Centre for the Study of the Social and Environmental Determinants of Nutrition, Population Health, Health Systems and Innovation Unit, Human Science Research Council, 12th Floor Plein Park Building, 69-83 Plein Street, Cape Town, 8001, South Africa; 2 UCT/MRC Research Unit for Exercise Science and Sports Medicine, Department of Human Biology, Faculty of Health Sciences, University of Cape Town, 3rd Floor, Sports Science Institute of South Africa, Boundary Road, Newlands, Cape Town, 7700, South Africa; 3 UCT/MRC Research Unit for Exercise Science and Sports Medicine, Medical Research Council, Tygerberg, Francie van Zijl Drive, Parowvallei, Cape Town, 7505, South Africa

## Abstract

**Background:**

International studies suggest ethnic differences in obesity prevalence may be due, in part, to differences in body image and body size dissatisfaction between groups. Further, there is evidence to suggest that there is a familial resemblance in body image between mothers and their younger (preadolescent) daughters. This research was therefore conducted to specifically identify the extent to which family status (presented as mother-daughter resemblance) and ethnicity impact on body image attitudes and perceptions of South African mothers and their pre-adolescent daughters.

**Methods:**

Mother-daughter dyads (n = 201, 31% black, 37% mixed ancestry and 32% white) answered questions regarding their body image perception (the way they saw their body size status), their body image ideals, and body image attitudes (body size dissatisfaction in particular, presented as the Feel-Ideal Difference [FID] index score). Mothers' and daughters' body image results were compared within dyads and across ethnic groups using repeated measures of ANOVA.

**Results:**

Overall, body image resemblances exist between South African mothers and their pre-adolescent daughters. Mothers and daughters chose similarly weighted silhouettes to represent their body size ideals (p = 0.308), regardless of their ethnicity or body mass index (BMI). The FID index scores were similar between mothers and their daughters only after the confounding effects of maternal BMI were removed (p = 0.685). The silhouettes chosen to represent thinness were also similar between mothers and their daughters (p = 0.960) regardless of ethnicity and maternal BMI. On the other hand, the silhouettes chosen to represent fatness were similar (p = 0.342) between mothers and their daughters, only after the confounding effects of maternal BMI were removed. Lastly, mothers and their daughters chose similarly weighted silhouettes as engendering feelings of beauty, respect and happiness (p = 0.813; p = 0.615 and p = 0.693, respectively). In this instance, black mother-daughter dyads chose significantly heavier silhouettes than the other ethnic groups. This implies that black mothers and daughters associate beauty, respect and happiness with a bigger body size.

**Conclusion:**

Resemblances exist between pre-adolescent girls and their mothers on issues related to ideal and attitudinal body image. In this regard, South African researchers should consider the effects ethnicity and family status on body image of women when developing targeted interventions to prevent or manage obesity.

## Background

Obesity is an important risk factor for chronic non-communicable diseases globally [1]. In South Africa, like many other developing countries, adult women are the most vulnerable group, with a markedly higher prevalence of obesity than men [2]. There is substantial evidence associating body image to women's response to weight changes and attitudes towards weight control [3-5]. In South Africa for example, women are less likely to see themselves as overweight, irrespective of body size [6,7]. In their study Puoane et al. [6] found that only 22.1% of South African women of all races perceived themselves as being overweight, when in fact 56.6% of women interviewed were classified as overweight and obese. These findings were influenced by ethnicity in that only 27% of overweight or obese black women correctly perceived themselves as overweight compared to 65% of mixed ancestry and 100% of white overweight or obese women. Similar results have previously been observed in urban South African adolescent girls in that black adolescent participants were less dissatisfied with their body size and shape, and were also less likely to desire a smaller body size than mixed ancestry and white girls [5,8].

Similar results have been observed in America in that, low-income African American women of different age groups tend to have fewer weight-related body image concerns than their white counterparts [9-12]. Similarly, Flynn and Fitzgibbon [12] showed that normal weight black adolescent girls have a preference for a larger body size compared to white adolescent American girls. Accordingly, one may surmise that the black adolescent population is less motivated than the white adolescent population to engage in behaviors that would prevent the development of obesity. In accordance with these findings, Stevens et al. [10], in their study comparing overweight white and black adult American women, found that black women were 40% less likely to feel guilty after eating, 2.5 times more likely to be satisfied with their weight and 2.7 times more likely to consider themselves attractive than white women. In this regard, the tolerance of a bigger body size status in black women appears to be the modifier of high self-esteem and positive body image. Moreover, in the same study, among those women who were not overweight, white American women perceived themselves to be larger in body size and reported lower ideal body weight compared to black American women [10].

While the previous studies argue strongly in favour of ethnic-specific differences in body size tolerance in women, the extent to which these differences may be attributed to intra-familial effects is not clear. Indeed, various international studies have previously shown that the family environment, particularly the mother-child relationship, has an influence on obesity risk [11-15]. In these studies, the relationship between mothers and daughters has been found to be stronger than that between mothers and sons. Further, the mother-daughter relationship has been shown to influence body image, with mothers unintentionally modelling both positive and negative body image to their daughters [13,15].

There is little research directed at exploring the socio-cultural factors influencing body size perceptions and attitudes between mothers and daughters in relation to obesity in countries undergoing epidemiological transition, such as South Africa. This may be considered in the context of the co-existence of maternal over-nutrition and childhood under-nutrition, which has been described in Africa [16].

In our previous study of 204 South African women and 333 pre-adolescent girls from different ethnic groups, we showed that overall, participants had appropriate perceptual body size, in that positive correlations were found between the silhouettes chosen by the participants to represent their 'feel' and their actual/measured body size and body fat percentage [3]. However, these relationships were altered by ethnicity, particularly with respect to body size tolerance in the girls, in that black girls had less body size dissatisfaction at higher actual BMI than white and mixed ancestry girls [3].

Therefore, the aim of this study was to identify the extent to which family status (presented as mother-daughter resemblance) and ethnicity, might explain differences or similarities in the body image and perceptions of South African mothers and their pre-adolescent daughters.

## Methods

### Study population

This study is a secondary analysis of body image data that forms part of a larger project where factors associated with obesity in 204 mothers and their 372 pre-adolescent daughters were studied [3]. To recruit participants, fifteen primary schools in the Cape Town Metropole Area were randomly selected and sampled on the basis of divergent socio-economic status (ranging from low, middle to high socioeconomic status). All girls (ages 9-12 yrs, grades 4-5) and their mothers were invited to participate in the study. The response rate was 89% for girls and 61% for mothers, such that 30% of girls interviewed were from schools within the highest socioeconomic stratum, 49% from the middle socioeconomic stratum and 21% from the lowest socioeconomic stratum.

To control for the potential confounding effects of diverging stages in sexual maturation on body weight, body fatness and body image, girls were also asked to estimate their pubertal stage. Self-rating has been found to be a reliable way to identify pubertal development in South African children [17]. Self-rating was done using diagrammatic sketches of Tanner (1962) classifications of breast development and pubic hair growth. Participants did this individually with privacy ensured by the interviewer. The girls' developmental stage was classified based on self-staging of both breast and hair development. Of the 372 girls who participated, 89% were in Tanner Stages 1, 2% in Stage 3 and 7% in Stage 4. Four percent of the girls could not correctly identify their stage of development. Only girls who were in Tanner Stages 1 and 2, and who returned to school with the signed consent forms (n = 333) were included in the final analysis, of whom 32% were black, 34% were of mixed ancestry and 34% were white. Of the 204 mothers and caregivers to the girls who responded, 31% were black, 37% were of mixed ancestry and 32% were white.

To further control for the potential confounding effects of diverging cultural/ethnicity beliefs within mother-daughter pairs, all mother-daughter pairs that were of mixed race (meaning those pairs comprised of mothers who were of different ethnicity to their daughters) were excluded. As such, a total sample included in this analysis consisted of 201 mother-daughter pairs (dyads), including 31% black, 37% mixed ancestry and 32% white girls whose mothers were of the same ethnicity as their daughters and had agreed to participate in the study. The research was approved by the Human Research Ethics Committee, Faculty of Health Sciences, University of Cape Town (REC REF NO: 185/2002); as well as the Department of Education in South Africa. Mothers signed the consent forms and also consented for their daughters to participate in the study.

### Body image perception and attitudes

Details and the validation procedures of the methods used to assess body image attitudes [feelings about body size status, defined as FID Index] and body image perception (the way women see their body size status and attitudes towards a smaller or bigger body size) for both girls and their mothers are previously described by Mciza et al. [3]. In brief, silhouettes were used to identify body size and shape status of the girls and their mothers. A set of eight silhouettes ranging from very thin to very heavy, which were derived from the Pathways Study [18], were redrawn with permission and modified to represent ethnic diversity in South Africa. These changes did not affect the original body sizes and shapes. Further, a set of eight silhouettes, adopted from the Stunkard's body image figures [19], also ranging from very thin to very heavy was used for mothers. These silhouettes were allocated numbers 1 to 8 from left to right and the numbers were used for comparative analyses. The silhouettes were grouped into four categories following the internationally successfully use of classification [20-22]: silhouettes 1, 2 and 3 equivalent to underweight (BMI ≤19.9 kg/m^2 ^or the World Health Organization (WHO) BMI ≤ 49.9^th ^percentile for mothers and daughters, respectively), silhouettes 4 and 5 represented normal weight (BMI = 20 - 24.9 kg/m^2 ^or WHO BMI = 50 - 84.9^th ^percentile for mothers and daughters, respectively), silhouettes 6 and 7 represented overweight (BMI = 25 - 29.9 kg/m^2 ^or WHO BMI = 85 - 94.9^th ^percentile for mothers and daughters, respectively) and silhouette 8 represented obesity (BMI ≥ 30 kg/m^2 ^or WHO BMI ≥ 95^th ^percentile for mothers and daughters, respectively).

To measure body image perception, each participant was shown the afore-mentioned sets of age-adjusted silhouettes and was asked to select the silhouette that she felt best represented her current weight. The silhouette selected indicated how she saw herself, presented as "Feel". The comparison between the "Feel" silhouette and the categories of BMI and body fat percentage was conducted and the differences were considered as a proxy of body image disturbance. Individuals were classified as correctly identifying their body size if they selected a silhouette that corresponded to their measured BMI and body fat percentage category. Whereas, participants who selected a silhouette that was higher than their measured BMI and body fat percentage category were classified as overestimating their body size. Moreover, individuals were classified as underestimating their body size when they selected a silhouette that was lower than their measured BMI and body fat percentage category.

### Measuring BMI and Body Fat

Body weight (for both mothers and daughters) was assessed in light clothing, without shoes, and recorded to the nearest 0.5 kg using a calibrated electronic scale (TANITA HD-309, Tanita Corporation of America Inc, USA). The height (for mothers only) was measured without shoes to the nearest 0.5 cm using a calibrated height meter. The BMI was calculated as weight (in kg) divided by the square of height (in m) for mothers; and the WHO BMI percentiles (weight-for-age) were determined for girls [22]. Triceps, biceps, subscapular and suprailiac skinfold thickness were measured (for both mothers and daughters) using calibrated Harpenden callipers, and recorded to the nearest 0.1 mm. Percentage body fat measurements were calculated using standard equations by Durnin and Womersely [23] for mothers and by Lohman [24] for girls.

To assess body image attitudes, FID Index scores were calculated using a procedure similar to Body Image Discrepancy scores created in the studies of Bulik et al. [25] and Fitzgibbon et al. [26] to assess body size dissatisfaction. The personal FID Index scores were created by determining the difference in the number of silhouette selected which best represented participants' current appearance 'Feel', and the one they thought was their 'Ideal' (representing the silhouette they preferred to look like). In addition, a 'friends' FID index score was calculated, based on the silhouette selected by the participants that they believed their friends regarded as 'Ideal'. When a participant selected a smaller sized 'Feel" silhouette than their 'Ideal' silhouette, a negative FID index score was obtained. A higher score represented greater body size dissatisfaction. On the other hand, a score closer to zero, represented less body size dissatisfaction.

To assess weight-related beliefs, previously validated fat belief constructs for both mothers and their daughters were used [3]. These constructs were comprised of questions asking whether a fatter woman or girl would feel better about herself; will feel more like a woman/girl; will be happier; will have more friends and become healthier, in comparison to a thinner woman/girl. Participants who gave affirmative responses to most of these questions demonstrated higher regard towards a bigger body size status. Whereas, participants who gave negative responses to the questions demonstrated less regard towards a bigger body size status.

Finally, participants were asked to identify silhouettes that they regarded as thin, normal weight and fat; as well as those showing respect, happiness and beauty. These adjectives were adopted from previous South African studies that examined issues related to the body image of women [27,28].

### Statistical analysis

All data were analyzed using Statistica (StatSoft, Tulsa, OK, USA version 7.0). Data were expressed as means ± standard deviations. Participants' characteristics by ethnic group were compared using analysis of variance (ANOVA). The effects of family status and ethnic group on the attitudinal and perceptual body image of participants were calculated using repeated measures ANOVA, and the results were then adjusted for maternal BMI. Family status (mother and daughter data) was used as the within-subject factor. Fischer's LSD post hoc test was used to evaluate the within ethnic group status. This method has previously been described by Ogden and Elder [14].

## Results

### Participants' characteristics

The characteristics of the 201 mother-daughter dyads are presented in Table 1. Black girls were significantly older than the white girls, but there were no ethnic differences in body composition between the girls. Conversely, the white women were significantly older than the mixed ancestry women. The black women had a significantly higher BMI and body fat percentage than the mixed ancestry and white women.

**Table 1:** Physical characteristic of the mothers and their daughters according to ethnicity

	Black (n = 44)		Mixed ancestry (n = 64)		White (n = 60)	
	Mothers	Girls	Mothers	Girls	Mothers	Girls
**Age (years)**	38.5 ± 9.0	10.5 ± 0.9^b^	38.4 ± 4.7^a^	10.1 ± 0.7	41.5 ± 4.6^a^	9.9 ± 0.8^b^
**BMI (kg/m**^**2 **^**women) and WHO BMI (% girls)**	32.1 ± 7.1^cd^	56.7 ± 32.8	26.1 ± 4.5^c^	58.8 ± 29.2	25.2 ± 4.0^d^	60.9 ± 25.5
**Body fat (%)**	34.7 ± 6.3^ab^	24.7 ± 5.3	32.4 ± 4.9^a^	24.3 ± 4.7	31.6 ± 4.8^b^	24.9 ± 4.4

Values are expressed as Means ± Standard Deviations: Matching superscripts present significant differences between groups: ^ab ^p < 0.05, ^c^^d^p< 0.001

### Family status influences on perceptual body size

Table 2 and 3 present the scores of silhouettes selected by both mothers and their daughters, representing different dimensions of body image. There was no observed within-family resemblance on the perceptual body image of the participants. Overall, mothers and daughters chose significantly different silhouettes reflecting their actual body size or 'Feel' image (5.0 ± 1.5 versus 4.2 ± 1.2, p < 0.001), with girls choosing a smaller body size silhouette than their mothers (Table 2). Further, the ethnic effect was displayed, such that the mother-daughter dyads from black families chose significantly bigger sized silhouettes to reflect their 'feel' than the other ethnic groups of mother-daughter dyads (p < 0.01) (Table 3). This was despite the fact that the actual/measured BMI was significantly different between ethnic groups of mothers, but not for the girls.

**Table 2:** Silhouettes chosen by mothers and their daughters to represent different dimensions of body image (intra-familial effect is present for constructs in which P values are > 0.05)

Silhouettes	Overall Mother-Daughter relationship (n = 166)	p value	p value after adjusting for maternal BMI
**Perceptual body size**	5.0 ± 1.5 vs. 4.2 ± 1.2	<0.001^1^	<0. 001^1^
**Ideal body size**	3.9 ± 1.0 vs. 3.7 ± 1.4	= 0.308^2^	= 0.308^2^
**Personal FID index score**	1.2 ± 1.4 vs. 0.5 ± 1.4	<0.001^1^	= 0.685^2^
**Friend FID index score**	1.2 ± 1.6 vs. 0.5 ± 1.6	<0.001^1^	<0.05 ^1^
**Thin figure**	1.3 ± 0.7 vs. 1.3 ± 0.9	= 0.96^2^	= 0.96 ^2^
**Normal figure**	3.9 ± 0.7 vs. 4.5 ± 1.2	<0.001^1^	<0.001 ^1^
**Fat figure**	8.1 ± 1.4 vs. 7.8 ± 0.8	<0.05^1^	= 0.342 ^2^
**Beautiful figure**	3.7 ± 0.9 vs. 3.7 ± 1.4	= 0.813^2^	<0.001 ^1^
**Respected figure**	4.4 ± 1.8 vs. 4.5 ± 2.0	= 0.615^2^	= 0.606 ^2^
**Figure showing happiness**	4.1 ± 1.9 vs. 4.1 ± 1.6	= 0.693^2^	<0.001 ^1^

Values are presented as Means ± Standard Deviations. ^1^p < 0.05 - no mother-daughter (family) resemblance; ^2^p > 0.05 - family resemblance present

**Table 3:** Ethnic differences in silhouettes chosen by mothers and their daughters representing dimensions of body image

	Ethnicity					
Silhouettes	Black (n = 44)		Mixed Ancestry (n = 64)		White (n = 60)	
	Mother	Girl	Mother	Girl	Mother	Girl
Perceptual body size*	5.5±1.9	4.7±1.5	5.0±1.3	4.0±1.0	4.7±1.2	4.1±1.1
**Ideal body size**^**#**^	4.4 ± 1.2	4.5 ± 1.8	3.8 ± 0.7	3.5 ± 1.1	3.5 ± 0.8	3.4 ± 1.1
**Personal FID index score**	1.1 ± 2.0	0.0 ± 2.1	1.2 ± 1.3	0.5 ± 1.1	1.2 ± 1.1	0.8 ± 1.1
**Friend FID index score**^**#**^	1.4 ± 2.2	0.03 ± 2.2	1.1 ± 1.4	0.5 ± 1.4	1.1 ± 1.2	0.9 ± 1.2
**Thin figure**	1.2 ± 0.7	1.5 ± 1.4	1.2 ± 0.5^3^	1.1 ± 0.4^3^	1.5 ± 0.8	1.2 ± 0.6
**Normal figure**	4.2 ± 1.2	5.2 ± 1.6	3.9 ± 0.6	4.2 ± 0.7	3.6 ± 0.6	4.1 ± 0.9
**Fat figure**	8.9 ± 0.4	7.6 ± 1.0	8.1 ± 1.3	7.9 ± 0.3	7.3 ± 1.7	7.8 ± 1.0
**Beautiful figure**^**†**^	4.0 ± 1.3	4.3 ± 1.8	3.8 ± 0.7	3.5 ± 0.9	3.4 ± 0.8	3.2 ± 1.1
**Respected figure**^**†**^	5.4 ± 2.4	5.3 ± 1.9	4.0 ± 1.5	4.1 ± 2.1	3.7 ± 1.0	4.1 ± 1.9
**Figure showing happiness**^**†**^	4.6 ± 1.9	4.2 ± 2.2	4.1 ± 1.8	4.2 ± 1.4	3.7 ± 1.8	3.9 ± 1.2

Values are presented as Means ± Standard Deviations. *p < 0.01 black families versus white families and families of mixed ancestry, ^**#**^p < 0.01 black versus white families, ^**†**^p < 0.001 black versus white families and families of mixed ancestry

### Family status influences on ideal body size, and attitudes towards body size status

When selecting the silhouette that represented their "Ideal" body size, there were no significant differences observed between mother-daughter dyads (p = 0.308). This suggested that there was an intra-familial resemblance for the so-called "ideal" body size (Table 2), despite the fact that black mothers-daughter dyads chose significantly heavier silhouettes than the other two ethnic groups (p < 0.01) (Table 3). This intra-familial effect remained even after adjusting for differences in maternal BMI.

Based on the FID index scores, girls were generally less dissatisfied with their body sizes, with significantly lower personal FID index scores than their mothers (0.5 ± 1.4 versus 1.2 ± 1.4, p < 0.001) (Table 2). These mother-daughter differences did not vary across ethnic groups (Table 3). However, after adjusting for maternal BMI, overall the mother-daughter dyads had similar FID index scores (p = 0.685). Further, mother-daughter dyads from black families demonstrated significantly less body size dissatisfaction than mother-daughter dyads from white families (p < 0.01).

The FID index scores attributed to the participants' friends were also lower in the girls compared to their mothers (0.5 ± 1.6 versus 1.2 ± 1.6, p < 0.001) (Table 2), however, there was a significant interaction effect, in that black girls perceived that their friends had a greater body size tolerance, than their mothers (Table 3). The scores remained lower in girls compared to their mothers even after adjusting for differences in maternal BMI (p < 0.05), and ethnic differences also remained (p < 0.001) with scores lowest in dyads from black families.

A 'fat belief' construct was formulated from the participants' association of fatness with certain attributes such as beauty, happiness, popularity, prosperity and health [3]. Girls had higher regard towards fatness or associated a larger number of positive attributes to body fatness than their mothers (4.5 ± 3.7 versus 1.7 ± 1.0, p < 0.01, data not shown). This pattern did not change even after adjusting for maternal BMI.

When participants were asked to identify silhouettes that they regarded as 'thin', 'normal' and 'fat', mothers selected similarly weighted silhouettes to those selected by their daughters for 'thinness' (p = 0.96) (Table 2). However, girls across all ethnic groups identified a larger silhouette as 'normal' (p < 0.001) and regarded a smaller silhouette as 'fat' (p < 0.05) compared to their mothers (Table 2). No ethnic differences were observed in this instance (Table 3). Adjusting for ethnicity did not change the pattern of the silhouettes selected as 'thin' and 'normal', but did change those of the silhouettes selected for 'fatness', with mothers choosing similar weighted silhouettes as their daughters (p = 0.342).

Lastly, participants were asked to identify silhouettes that they regarded as beautiful, showing respect and happiness from the sets of 8 age-adjusted silhouettes. Girls across all ethnic groups identified similarly weighted silhouettes to those identified by their mothers as beautiful, showing respect and happiness (p = 0.813, p = 0.615 and p = 0.693, respectively) (Table 2). Further, black mother-daughter dyads chose significantly heavier silhouettes than the other two ethnic groups (p < 0.001) (Table 3). However, the pattern changed for the silhouettes selected as showing beauty and happiness, after adjusting for maternal BMI (p < 0.001 and p < 0.001, respectively). In this instance, white girls chose smaller silhouettes to represent beauty and happiness than their mothers. Black girls on the other hand chose larger silhouettes to represent beauty and happiness than their mothers, whereas similarities still remained within the mixed ancestry families.

## Discussion

This study provides novel insight into the important and respective roles of family membership or maternal modelling and ethnicity on different dimensions of body image such as body size perception, and body size dissatisfaction in women and their daughters from South Africa. Intra-familial resemblance for perceived body size, ideal body size and body size dissatisfaction were found when the potential confounding of maternal body size was removed. In addition, mothers and daughters from black families demonstrated an overall greater body size tolerance than their white and mixed ancestry counterparts. To our knowledge, this is the first time in South Africa that these two constructs, family membership and ethnicity, have been compared in the same population, with respect to different dimensions of body image in relation to obesity. These results corroborate those of international studies [29,30], suggesting intra-familial resemblances in body image between mothers and their pre-adolescent daughters. Further, these results corroborate both local and international results that have shown a greater tolerance for a bigger body size in women of African descent, compared to other cultures [4,7,8,10-12,26,31].

Striking findings from this study were that black girls seemed to differ from their mothers in terms of body size preference, which contrasts to the findings of Hill and Bhatti [30]. In this study, black girls were leaner, yet they preferred a larger silhouette. On the other hand, the majority of black mothers were obese, yet they preferred leaner silhouettes. What is of concern is that South Africa studies suggest that overweight black women are resistant to adopt health behaviours, despite knowing that they are at an increased risk for non-communicable diseases [28,32-34]. This resistance may be partly endorsed by the weight-loss stigma associated with the HIV/AIDS wasting syndrome [34], and the fact that, being overweight in the black culture is a symbol of wealth, autonomy, attractiveness and happiness [16,27,28,32,33].

Indeed, black women participating in the current research were more likely to regard fatness as a sign of health, beauty, respect and happiness. Similarly, other South African studies [16,27,28,32,33] have shown that in the black communities of South Africa, being overweight is desirable as it denotes beauty, happiness and affluence. Further, Matoti-Mvalo [34] reported that these beliefs are now further exacerbated by the idea that being thin can be equated with HIV/AIDS virus infection. The following quote typifies this: 'If you are thin, people think that you are sick -- you may have TB or HIV/AIDS' [32,34]. Based on these beliefs structures, black South African women may be more reluctant to lose weight than women of different ethnic origin. Interestingly, white and mixed ancestry families participating in the current research only associated a leaner body size with beauty, health and happiness. Indeed, Brink [35] has highlighted that in the Western culture, thinness does not just mean the size of the body, but thinness is associated with qualities of being healthy, attractive and in control. In contrast, a fat body is viewed as a sign of poor health, laziness, sloppiness and lack of personal will [35,36].

Research internationally has also shown that the social stigma associated with being obese is more prevalent in women than men, and women are more likely to be discriminated against because of their weight [37]. Some of the social stigmas attached to obesity relate to the attributes and cultural emphasis placed on appearance and especially women's body size [37]. In the current research it has been clearly shown that girls perceived that their friends were more tolerant of their body size than their mothers. The most likely explanation for these differences may be that the girls, on average, were within the normal range for expected weight for height, compared to the maternal cohort, who had a mean BMI of 27.8 kg/m^2^. This is in line with studies which show that self-esteem tends to be higher and body size dissatisfaction lower in children who are within the normal range of body weight [38,39]. In accordance with other studies [8,39] ethnicity modulated these effects, with the perception of body size tolerance amongst friends and peers being greater in black girls and their mothers, compared to other ethnic groups. Young-Hyman et al. [40] also suggested that body size acceptance by the primary caregivers of children may also be influenced by cultural differences. Indeed, Puoane et al. [5] recently highlighted the ambiguity concerning overweight and obesity in urban South African girls from black families in which two-thirds of those surveyed associated obesity with happiness and wealth and the remainder were ambivalent. Thus, in terms of societal norms, current body size status and culture may influence both one's individual body size satisfaction, as well as one's perception of how they are viewed by others.

In summary, this study has shown that despite South African preadolescent girls being significantly less dissatisfied about their body size compared to their mothers, intra-familial resemblances for body size dissatisfaction existed when the potential confounding differences in maternal body size were removed. Further, this study has highlighted that society and culture mediates body size dissatisfaction. These results have important implications for the development of obesity in South Africa, given the high prevalence of obesity in women, which also differs between ethnic groups. This strongly suggests that in South Africa health promotion needs to be ethnic-specific, and should always involve families, not individuals. Strategies and interventions should be directed at increasing the awareness of a healthy body size status and maintaining it in an attempt to prevent obesity. This is highly important for those populations that are at the highest risk of becoming overweight and obese, but may not be bothered when they are overweight, black South African women in particular.

There are a number of limitations to this study. Firstly, the mothers and their daughters' eating and exercising behaviours and attitudes were not analysed, as this was beyond the scope of this paper. However, this is of relevance as international studies have shown that family environment and parental modelling influence a child's dietary intake, diet quality and participation in exercise activities [11,12,41]. Secondly, we only included preadolescent girls. The inclusion of adolescent girls may have provided further insight regarding children's attitudes and perceptions towards body image, which has been shown to be influenced by children's sexual maturity. Future studies including this group are encouraged.

## Conclusions

To conclude, the findings from the this study suggest that South African researchers, educators and health promoters should consider the effects of family environment and ethnicity on body image, when developing intra-personal and targeted interventions for the prevention and management of obesity. Most particularly, health education should not only be directed to the affected (the overweight/obese), but also include the whole family, so as to help dispel the myth and stereotypes suggesting "big" to be beautiful, healthy and respected. More focus is to be directed to those under-served and vulnerable communities, young black South African pre-adolescent children, in particular, who are at risk of developing to be overweight adults.

## Competing interests

The authors declare that they have no competing interests.

## Authors' contributions

ZJM - conducted literature search, conceptualized the information in the paper, produced the first draft of the paper, made all the changes resulting to editing by co-authors and finalised the paper; JHG -edited the paper; VEL- helped in the conceptualization of the paper, the statistical analysis, the review of the literature and edited all the drafts of the paper.

All authors have read and approved the final manuscript.

## Pre-publication history

The pre-publication history for this paper can be accessed here:

http://www.biomedcentral.com/1471-2458/11/433/prepub
